# Perioperative nursing care for patients with fibrosing mediastinitis-associated pulmonary hypertension undergoing percutaneous intervention

**DOI:** 10.3389/fcvm.2026.1801542

**Published:** 2026-09-10

**Authors:** Ya He, Xue-hua Cao, Jia-lan Xu, Wen-ting He, Li Xiao, Qin Lv

**Affiliations:** 1 Heart, Lung and Vessels Center, Sichuan Academy of Medical Sciences & Sichuan Provincial People’s Hospital, Affiliated Hospital of UESTC, Chengdu, Sichuang, China; 2 Department of Gynecology, Sichuan Academy of Medical Sciences & Sichuan Provincial People’s Hospital, Affiliated Hospital of UESTC, Chengdu, Sichuang, China; 3 Department of Geriatric Cardiology Nursing, Sichuan Academy of Medical Sciences & Sichuan Provincial People’s Hospital, Affiliated Hospital of UESTC, Chengdu, Sichuang, China; 4 Department of Respiratory and Critical Care Medicine, Sichuan Academy of Medical Sciences & Sichuan Provincial People’s Hospital, Affiliated Hospital of UESTC, Chengdu, Sichuang, China

**Keywords:** complications, fibrosing mediastinitis, hemodynamic improvement, interventional therapy, percutaneous transluminal pulmonary angioplasty, perioperative nursing, pulmonary hypertension, rehabilitation

## Abstract

**Objective:**

To summarize the perioperative nursing experience of patients with pulmonary hypertension caused by fibrosing mediastinitis (PH-FM) undergoing interventional therapy, and to provide evidence-based guidance for clinical nursing practice.

**Methods:**

A total of 54 PH-FM patients admitted to our center from May 2024 to August 2025 were treated with pulmonary vascular interventional therapy, and a comprehensive perioperative nursing protocol was implemented, encompassing, including preoperative assessment and preparation, intraoperative monitoring and intraoperative collaboration, postoperative complication prevention, and rehabilitation guidance.

**Results:**

All 54 patients successfully completed the procedure with no procedural mortality. Only 25 patients with pulmonary artery stenosis and 19 patients with pulmonary vein stenosis had complete paired hemodynamic data. The results revealed that the mean trans-stenotic pressure gradient of the pulmonary artery decreased by 27.64 mmHg, and that of the pulmonary vein decreased by 21.89 mmHg, with significant improvements in hemodynamic indicators. Intraoperative complications included 5 cases of hemoptysis, 7 cases of cough, 2 cases of vasovagal reactions, and 1 case of chest tightness; postoperative complications included 1 case of lung injury and 1 case of hematuria, all of which were managed promptly. All patients were discharged in stable condition 4–11 days after procedure. During follow-up, the restenosis rate was 9.30% for pulmonary artery lesions and 22.73% for pulmonary vein lesions.

**Conclusion:**

Comprehensive and individualized perioperative nursing care for PH-FM patients receiving interventional procedures is associated with early identification and management of complications, favorable procedural safety and improved patient rehabilitation, showing promising correlations with better clinical outcomes in routine care.

## 1 Introduction

Fibrosing Mediastinitis (FM) is a rare disease characterized by excessive proliferation of fibrous tissue in the mediastinum. Progressive replacement of normal mediastinal adipose tissue by proliferating fibrous tissue characterizes the disease process, which then compresses mediastinal structures such as the bronchi, esophagus, blood vessels, and heart (1). FM is mostly triggered by infectious or non-infectious inflammatory etiologies: the former includes infections with pathogens such as Histoplasma capsulatum and Mycobacterium tuberculosis, while the latter includes diseases such as sarcoidosis (2). Geographically, FM in the United States and temperate regions is often associated with Histoplasma capsulatum infection (3), whereas in China, the most common cause is tuberculosis (TB) infection (4). Epidemiological data reveal a substantial burden of latent tuberculosis infection (LTBI) in China, with over 740,000 new TB cases reported in 2023 and an incidence rate of 5,200 per million population (5). This suggests a considerable potential risk of TB-associated FM in this region. The infiltrative and compressive effects of proliferating fibrous tissue in FM on pulmonary blood vessels can lead to vascular stenosis and subsequent pulmonary hypertension (PH). With disease progression, right heart failure and even death may occur. Studies have shown that PH caused by FM (PH-FM) has a high rate of misdiagnosis and underdiagnosis (6), resulting in most patients not receiving timely and effective treatment and thus having a poor prognosis. In recent years, with the continuous advancement of percutaneous pulmonary vascular interventional therapy (7), percutaneous transluminal pulmonary angioplasty (PTPA) has shown significant efficacy in improving PH-FM-related symptoms and has become the preferred clinical treatment for this disease (8). However, such interventional procedures are complex, technically challenging, and associated with a relatively high incidence of complications and certain risks. Comprehensive perioperative nursing management is a critical determinant of procedural success and postoperative recovery, which is crucial for avoiding severe complications induced by interventional therapy and promoting postoperative rehabilitation. This study aimed to summarize perioperative nursing experience, complications and clinical outcomes in PH-FM patients undergoing interventional therapy, analyze correlations between standardized nursing interventions and clinical outcomes, and offer evidence for clinical nursing.

## 2 Materials and methods

### 2.1 Study population

This study was conducted as a single-center, retrospective observational cohort study of consecutive patients with fibrosing mediastinitis-associated pulmonary hypertension (FM-PH) who underwent percutaneous pulmonary vascular intervention at Sichuan Provincial People's Hospital between May 2024 and August 2025. All follow-up surveillance was completed by December 2025. The scheduled follow-up visits were arranged at 1, 3, 6 and 12 months after the procedure, and a centralized follow-up assessment of all enrolled patients was conducted in December 2025. Inclusion Criteria: (1) Patients diagnosed with fibrosing mediastinitis (FM) via contrast-enhanced chest computed tomography (CT) or magnetic resonance imaging (MRI): imaging revealed focal or diffuse fibrous tissue replacing mediastinal adipose tissue (predominantly at the pulmonary hilum), with or without punctate or dense calcification, accompanied by compression of adjacent mediastinal structures; tumors and other alternative diseases were excluded. Patients presented clinical manifestations and imaging changes consistent with pulmonary hypertension (PH), and FM-PH was further confirmed by right heart catheterization. (2) Patients with indications for percutaneous pulmonary angioplasty who received this interventional procedure. Exclusion Criteria: (1) Severe liver, renal, cardiac or hematological dysfunction that cannot tolerate interventional therapy. (2) Active pulmonary infection, massive hemoptysis or coagulation dysfunction before operation. (3) Combined malignant tumors or other severe life-limiting organic diseases.

### 2.2 Treatment methods

Pulmonary artery stenosis treatment: Patients were placed in the supine position, with the right femoral vein as the vascular access. Under local anesthesia, Swan-Ganz catheterization was performed to obtain hemodynamic data. Selective pulmonary angiography was then conducted to locate the stenotic lesions. After measuring the trans-stenotic pressure gradient, balloon angioplasty was performed using an appropriately sized balloon. Stent implantation was performed in cases of significant residual stenosis or recoil. Post-interventional angiography and pressure measurement were conducted to confirm technical success.

Pulmonary vein stenosis treatment: After right heart catheterization, transseptal puncture was performed to access the left atrium. The catheter was advanced to the target pulmonary vein for selective venography and pressure measurement. Staged balloon dilation was performed on the stenotic segment. Stent implantation was performed if necessary, followed by repeated pressure assessment to evaluate hemodynamic outcomes.

### 2.3 Perioperative nursing framework and intervention measures

#### 2.3.1 Preoperative nursing

The perioperative nursing team conducted a systematic assessment immediately after the patient's admission, focusing on cardiopulmonary function, oxygenation level, volume load, and past medical history. For patients on long-term diuretic therapy, electrolyte monitoring and volume status assessment was intensified to reduce the risk of perioperative hemodynamic fluctuations. Standardized oxygen therapy was provided to patients with hypoxemia to improve their preoperative baseline status. Given the abnormal pulmonary vascular structure and high risk of hemoptysis in PH-FM patients, the nursing team conducted prioritized preoperative hemoptysis risk assessments and developed individualized emergency plans in collaboration with physicians. For high-risk patients, resuscitation equipment and pharmacological agents were proactively prepared preoperatively to shorten the response time for complication management.

Meanwhile, preoperative health education was carried out through various forms to inform patients and their families about the surgical process, potential risks, and possible discomfort during the operation, with emphasis on the importance of timely reporting of symptoms. The patient's anxiety scores were assessed using the Generalized Anxiety Disorder Scale (GAD-7) (9). Psychological intervention and sedative support were provided to patients with significant anxiety or sleep disorders to improve their intraoperative cooperation.

#### 2.3.2 Intraoperative nursing

Continuous monitoring of electrocardiogram, blood pressure, and blood oxygen saturation was performed during the operation, with close observation of respiratory status and subjective discomfort. By maintaining communication with patients, nurses promptly identified early abnormal manifestations such as cough, chest distress, hemoptysis, and vagal reflex, and assisted physicians in adjusting the procedural pace or implementing interventions immediately. During the interventional procedure, the nursing team participated in the preparation of instruments and medications in advance and maintained a high degree of coordination with the operator to ensure the continuity and safety of the interventional operation. Through dynamic monitoring and rapid response, the risk of severe complication progression was mitigated.

#### 2.3.3 Postoperative nursing

After returning to the ward, patients received continuous monitoring, with a focus on changes in vital signs and oxygenation status. Standardized compression and fixation were applied to the puncture site, the Numerical Rating Scale (NRS) was used to assess patients’ pain (10), and regular assessments were conducted for bleeding, hematoma, or limb circulation disorders. Patients gradually resumed activities according to their condition to prevent thrombosis.

**Figure 1:**
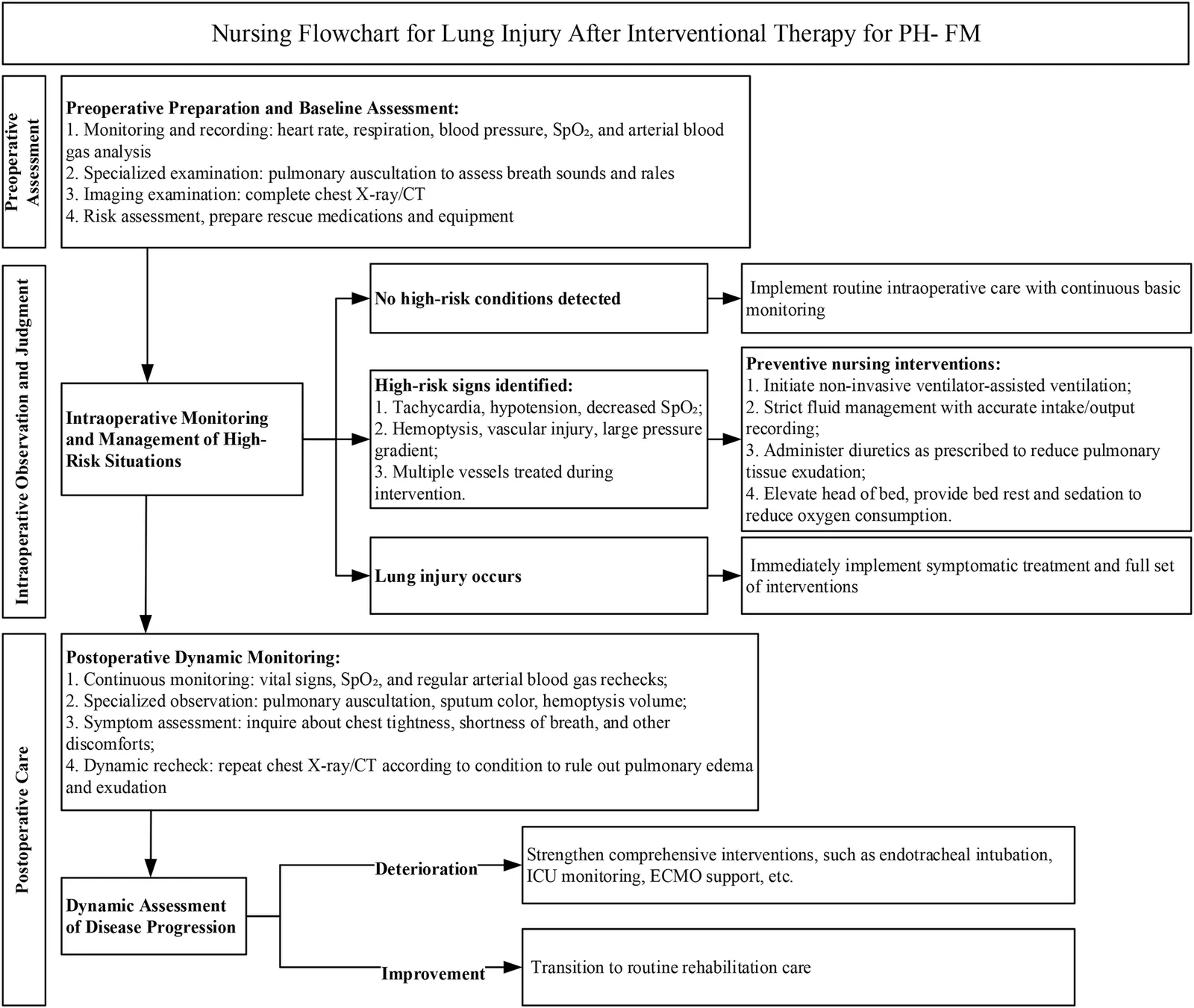
Nursing flowchart for lung injury after interventional therapy for PH-FM.

Notably, patients with PH-FM who receive interventional therapy may develop various intraoperative and postoperative adverse events (i.e., complications), including hemoptysis, lung injury, vagal reflex, puncture site pain, pulmonary vascular restenosis, and so on. Timely identification and targeted nursing care are required for these conditions. Reperfusion pulmonary edema (RPE) is a critical complication following PTPA, commonly caused by reperfusion pulmonary edema and intraoperative vascular injury. Patients after interventional therapy required close observation for at least 48 h, including continuous monitoring of vital signs, auscultation of lung breath sounds to assess respiratory function, and dynamic tracking of imaging results such as chest CT. By comparing preoperative and postoperative imaging features, potential risks were identified early to detect lung injury promptly. When lung injury occurred, nurses collaborated with physicians to implement non-invasive mechanical ventilation and provided ventilator-related nursing care. For patients with hemoptysis, maintaining airway patency was crucial: negative pressure suction devices, emergency medications, and supplies were kept at the bedside to promptly clear sputum, blood, and other foreign bodies from the oral cavity and airway. Patients were instructed on body positioning and anti-asphyxia measures during hemoptysis. In case of massive hemoptysis, emergency treatment was initiated immediately. If the patient showed signs of asphyxia, the body position was adjusted to a trendelenburg position to promote the discharge of accumulated blood in the airway, high-flow oxygen was administered, and a negative pressure suction device was used to quickly clear the airway. If asphyxia persisted after the above measures, tracheal intubation or tracheotomy was assisted immediately, followed by further life support. To address the potential lung injury induced by interventional therapy for PH-FM, a dedicated nursing flowchart has been developed to ensure timely detection and effective management of postoperative complications (Figure 1). Postoperatively, pain assessment and comfort care were also emphasized: non-pharmacological interventions were used to relieve discomfort at the puncture site, reducing vagal reflex induced by pain or tension.

In addition, psychological intervention was actively carried out postoperatively to encourage patients to participate in rehabilitation training, join patient associations or PH associations, and maintain an optimistic and positive mood. Internet resources were provided, such as participation in chronic disease management programs, transitional care, and “digital health platforms”. Continuous contact was maintained with professional diagnosis and treatment centers, and nurses acted as liaisons during patient referral to enhance the psychological well-being of patients and their families.

#### 2.3.4 Discharge guidance

Before discharge, nurses provided systematic health education to patients, including medication adherence, self-monitoring of symptoms, activity and dietary guidance, and follow-up arrangements. Studies have shown (11) that PTPA and stent implantation can treat pulmonary vascular stenosis caused by fibrosing mediastinitis, but postoperative restenosis is common, especially in pulmonary veins with a restenosis rate of 20%–50% (12). Given that some patients require staged or repeated interventional therapy, the importance of long-term follow-up was emphasized, and family members were instructed to participate in disease management to improve treatment continuity and safety. Rehabilitation exercise was carried out in a gradual manner, recommending a combination of aerobic exercise, resistance training, and breathing exercises. Home oxygen therapy was required for patients with hypoxemia.

### 2.4 Statistical methods

SPSS 26.0 software was used for statistical analysis. Measurement data conforming to normal distribution were described as mea*n* ± standard deviation (±s), and paired-samples t-test was adopted for intragroup comparisons before and after surgery. Non-normally distributed measurement data were presented with median and interquartile range, and the Wilcoxon signed-rank test was used for intragroup paired comparisons. The Shapiro–Wilk test was applied to assess the normality of data, with the test level set at *α* = 0.05. Enumeration data were described by frequency and percentage. A *P* value less than 0.05 was considered statistically significant.

## 3 Results

A total of 54 PH-FM patients caused by fibrosing mediastinitis were enrolled, including 23 males and 31 females, with a mean age of 64.24 ± 8.44 years. The mean pulmonary arterial pressure (mPAP) measured by right heart catheterization was over 20 mmHg. According to the classification of pulmonary vascular stenosis induced by fibrosing mediastinitis (13), type I (arterial type) accounted for 3.70% (2 cases) and type III (mixed type) accounted for 96.30% (52 cases). Other comorbidities and underlying diseases are shown in Table 1. Among the 54 patients, 34 initially underwent interventional therapy for pulmonary artery stenosis, and 20 initially received pulmonary vein stenosis management. 25 patients received staged hospital admissions for the treatment of pulmonary vascular stenosis: 9 cases were treated for pulmonary vein stenosis prior to pulmonary artery lesions; 13 cases underwent staged treatment for isolated pulmonary artery stenosis; 2 cases received treatment for pulmonary artery stenosis first and then pulmonary vein stenosis; and 1 case underwent staged intervention for pulmonary vein stenosis alone. The average length of hospital stay was 9.45 days. Most patients presented with moderate preoperative anxiety, with a mean GAD-7 score of 11.46 ± 2.38. In particular, patients who had undergone multiple surgeries experienced anxiety due to concerns about postoperative outcomes. For these individuals, we delivered targeted psychological interventions and detailed health education regarding treatment protocols.

**Table 1:** Baseline clinical characteristics of PH-FM patients (*n* = 54).

Item		Value
Number of cases		54
Age (years)		64.24 ± 8.44
Female[n (%)]		31 (57.41)
Type[n (%)]		
	Arterial (Type I)	2 (3.70)
	Mixed (Type III)	52 (96.30)
Hemodynamics（mmHg，$\bar{x}$±s）		
	PASP	63.59 ± 19.87
	PADP	23.57 ± 7.71
	mPAP	36.91 ± 11.83
Comorbidities[n (%)]		
	Bronchial stenosis	49 (90.74)
	Pericardial effusion	21 (38.89)
	Pleural effusion	27 (50.00)
	Atelectasis	18 (33.33)
	COPD	39 (72.22)
	Hypertension	28 (51.85)
	Diabetes Mellitus	11 (20.37)
	Coronary Artery Disease	1 (1.85)
	Chronic Pulmonary Heart Disease	24 (44.44)
	Hyperlipidemia	12 (22.22)
	Hyperuricemia	18 (33.33)
Past Medical History[n (%)]		
	Tuberculosis	8 (14.81)
	Pulmonary Embolism	1 (1.85)
GAD-7 score		11.46 ± 2.38

All 54 patients successfully completed the interventional procedure without any procedure-related deaths. Among patients who underwent their first interventional procedure, complete paired pre- and post-intervention hemodynamic data were collected and documented for 25 patients with pulmonary artery stenosis and 19 patients with pulmonary vein stenosis. The remaining patients were excluded from the analysis because complete paired pre- and post-procedural pressure gradient data could not be obtained due to intraoperative measurement constraints and incomplete hemodynamic records. The results revealed that the mean trans-stenotic pressure gradient of the pulmonary artery decreased by 27.64 mmHg, and that of the pulmonary vein decreased by 21.89 mmHg, with significant improvements in hemodynamics (Table 2). During the operation, 5 patients developed obvious cough and blood-tinged sputum during balloon dilation and stent deployment. The treatment was immediately stopped, and dilation or stent deployment was resumed gradually after the patient recovered. After the operation, the pressure gradient at the pulmonary vascular stenosis site was significantly reduced. To prevent postoperative reperfusion pulmonary edema, non-invasive ventilator-assisted ventilation was used, which was discontinued after no abnormal pulmonary signs were observed.

**Table 2:** Comparison of hemodynamic pressure gradients across vascular stenosis before and after procedure.

Item	Pre-op	Post-op	Difference	t-value	*p*-value
Pressure Gradient across PA Stenosis（mmHg，$\bar{x}$±s）	41.40 ± 20.92	13.76 ± 11.91	27.64 ± 19.04	7.259	0.001
Pressure Gradient across PV Stenosis（mmHg，$\bar{x}$±s）	24.17 ± 9.36	2.26 ± 4.00	21.89 ± 8.77	10.885	0.001

Statistical analysis was restricted to patients with complete paired pre- and post-intervention hemodynamic data obtained at the first interventional procedure. (*n* = 25 for PA; *n* = 19 for PV). PA, Pulmonary Artery; PV, Pulmonary Vein.

In terms of complications. Two patients developed nausea, cold sweats, and hypotension during the operation. Dopamine and atropine were injected intravenously immediately, combined with intravenous fluid replacement and norepinephrine for blood pressure elevation. After intervention, the patient's blood pressure fluctuated between 120 and 160/80–100 mmHg, heart rate between 80 and 100 beats per minute, and blood oxygen saturation between 90%–92%, with relieved symptoms. One patient reported chest distress during the operation, which was alleviated after symptomatic treatment with oxygen inhalation, furosemide, dexamethasone, etc. Seven patients developed cough during the operation, and the operation was suspended temporarily and resumed after the patient recovered. Postoperatively, 1 patient developed hematuria, which stopped after hemostatic treatment with posterior pituitary injection and carbazochrome. One patient reported chest distress postoperatively, with a measured left atrial pressure of 20/6 mmHg, which was considered acute left atrial failure. The patient's chest tightness was relieved after intravenous furosemide and oxygen inhalation, and the left atrial pressure was remeasured to 17/7 mmHg. In addition, 1 patient developed lung injury postoperatively: chest CT showed no pulmonary exudation preoperatively and 24 h postoperatively, but 36 h after PTPA, the patient suddenly developed pink frothy sputum and peripheral oxygen saturation (SpO2) dropped to 87%. The symptoms gradually relieved after intravenous injection of 20 mg furosemide and oxygen inhalation. Chest CT was rechecked 10 h later, confirming pulmonary exudation in the area supplied by the pulmonary artery with stent implantation, and non-invasive positive pressure ventilation was used to treat pulmonary edema. Approximately 92 h postoperatively, pulmonary exudation improved significantly (Table 3). All patients received elastic bandage compression and immobilization at the femoral vein puncture site for 6–8 h, and the compression was removed after no bleeding was observed. The mean postoperative NRS score of all patients was 3.06 ± 0.84. Physical interventions were implemented in 10 patients to relieve pain and discomfort. All patients were discharged with clinical improvement.

**Table 3:** Intraoperative and postoperative complications (*n* = 54).

Complication Type	Number of Cases	Incidence (%)	Management	Outcome
Intraoperative				
Hemoptysis	5	9.26	Pause procedure + Hemostatic drugs	Symptoms resolved, procedure completed
Cough	7	12.96	The operation was suspended temporarily	Symptoms resolved, procedure completed
Vasovagal Reaction	2	3.70	Dopamine + Atropine + Fluid resuscitation	BP/HR normalized
Chest Discomfort	1	1.85	Oxygen + Furosemide + Dexamethasone	Symptoms resolved
Postoperative				
Pulmonary Injury	1	1.85	Furosemide + Non-invasive PPV	Pulmonary infiltration improved
Hematuria	1	1.85	Pituitrin + Carbazochrome	Bleeding stopped
Chest Discomfort	1	1.85	Oxygen + Furosemide	Symptoms resolved

PPV, positive pressure ventilation.

All 54 patients received regular and continuous follow-up after discharge. One patient died of other comorbidities 23 days after discharge, and no patients were lost to follow-up. Median follow-up duration was 12.62 months, and the observation window spanned 1–19.43 months. Restenosis was defined as luminal stenosis greater than 50% in the target operative vessel identified by CTA or pulmonary angiography during follow-up, and the time of restenosis onset was recorded. Restenosis analysis was patient-based. Patients who received treatment for pulmonary artery stenosis were assigned to the pulmonary artery stenosis group, and those who received treatment for pulmonary vein stenosis were assigned to the pulmonary vein stenosis group. Patients receiving both treatments were included in both groups simultaneously. Among the followed patients, restenosis occurred in 4 patients with pulmonary artery stenosis, with a re-stenosis rate of 9.30%, and the onset time of restenosis was 3.88 ± 1.80 months postoperatively. Restenosis was observed in 5 patients with pulmonary vein stenosis, with a restenosis rate of 22.73%, and the onset time was 4.58 ± 1.65 months postoperatively. See Table 4.

**Table 4:** Incidence of postoperative vascular restenosis.

Group	Patients (n)	Cases with restenosis (n)	Restenosis rate (%)	Onset time of restenosis (month)
Pulmonary artery stenosis	43	4	9.30	3.88 ± 1.80
Pulmonary vein stenosis	22	5	22.73	4.58 ± 1.65

Restenosis analysis was patient-based. Patients receiving treatment for pulmonary artery stenosis were included in the pulmonary artery stenosis group, and those receiving treatment for pulmonary vein stenosis were included in the pulmonary vein stenosis group. Patients who received both treatments may be counted in both groups; thus, the two groups are not mutually exclusive.

## 4 Discussion

### 4.1 Impact of comprehensive perioperative nursing on procedural safety

Compared with interventional therapy for other types of pulmonary hypertension, interventional therapy for PH-FM is more complex, usually involving multiple procedures such as right heart catheterization, transseptal puncture, pulmonary arteriovenous angiography, percutaneous balloon pulmonary arteriovenous angioplasty, and pulmonary arteriovenous stent implantation (11). These procedures are not only time-consuming and technically demanding for operators but also associated with high surgical difficulty and multiple risks, making them a clinically challenging type of treatment. In this study, comprehensive preoperative vital sign monitoring and individualized nursing interventions were associated with the smooth conduct of the interventional procedures. The focus of intraoperative nursing was real-time monitoring and rapid response: through close observation of the patient's vital signs, nurses could detect early signs of complications promptly. In addition, active communication and psychological support with patients during the operation reduced their fear and enhanced treatment confidence.

In terms of specialized nursing, preoperative emphasis was placed on hemoptysis risk assessment and preoperative preparation. By comprehensively evaluating the patient's medical history, high-risk factors were identified in advance and corresponding emergency plans were developed. After improving preoperative stratified assessment of hemoptysis risk and equipment preparation, all 5 patients who developed hemoptysis during the operation received timely hemostatic treatment and successfully completed the procedure. Meanwhile, preoperative health education and psychological counseling helped patients better understand the surgical process, alleviate anxiety, and improve intraoperative cooperation. During the operation, close monitoring of the patient's vital signs and subjective complaints allowed 2 patients with vagal reflex to have relieved symptoms after intervention such as blood pressure elevation and fluid replacement, without severe adverse events. Postoperative nursing focused on early identification and intervention of complications, especially high-risk issues such as lung injury and hemoptysis (14), through close observation and dynamic tracking. Meanwhile, the combination of pain management and psychological intervention not only relieved the patient's physical discomfort but also promoted psychological rehabilitation. These findings suggest that enhanced perioperative nursing interventions were associated with the safety of interventional procedures, with low surgical risks and satisfactory patient safety and comfort observed in this cohort.

### 4.2 Clinical value of targeted nursing for complications

The main postoperative complications of PH-FM patients include lung injury (especially lung injury) and hemoptysis (14). In this study, preoperative hemoptysis risk assessment was improved, and within 48 h postoperatively, 1 case of lung injury was identified early through lung auscultation and dynamic imaging monitoring. The patient recovered after treatment with diuretics and non-invasive positive pressure ventilation. For puncture site pain, physical intervention measures relieved discomfort in 10 patients (pain score: 2–4 points), with no stress-induced complications caused by pain. In addition, patients with pulmonary artery or pulmonary vein stenosis face a considerable risk of postoperative re-stenosis. In our study, the re-stenosis rate was 9.30% for pulmonary artery stenosis and 22.73% for pulmonary vein stenosis. Previous studies have reported that the re-stenosis rate of pulmonary vein stenosis ranges from 20% to 50% (15), the importance of regular follow-up and medication adherence was emphasized in discharge guidance. Twenty-five patients undergoing staged treatment received reintervention promptly through follow-up, which further demonstrated the correlation of targeted nursing with complication control and long-term management.

### 4.3 Synergistic effect of multi-dimensional nursing system

Given that most PH-FM patients require multiple hospitalizations and interventional surgeries, they face significant mental stress and economic burden (16). The bio-psycho-social nursing model constructed in this study was correlated with favorable patient prognosis, which was fully demonstrated by our clinical outcomes. From the biological dimension, comprehensive preoperative risk assessment and real-time intraoperative monitoring were associated with timely management of all complications, including 5 cases of hemoptysis and 1 case of lung injury, achieving a 100% surgical success rate. In terms of the psychological dimension, preoperative psychological counseling and health education were associated with good intraoperative cooperation, and all 54 patients successfully completed the procedure. Postoperative psychological intervention and access to online resources were associated with reduced post-discharge anxiety and stronger confidence in long-term treatment. For patients with multiple hospital admissions, continuous psychological care was associated with sustained positive mental status and lower anxiety and depression related to long-term treatment (17). Among the 54 enrolled patients, only one patient died, and no others were lost to follow-up. From the social dimension, family and social supportive nursing was associated with better access to rehabilitation resources and higher compliance with postoperative rehabilitation training. Notably, the 54 patients in this cohort were from multiple regions of China, where medical insurance reimbursement policies vary across areas. These discrepancies created unequal financial pressure for patients undergoing repeated staged surgeries and long-term follow-up. The involvement of family and social support provided patients with practical economic and emotional support, enabling them to better cope with various challenges during treatment (18). Nurses delivered individualized family health education and transitional nursing services in accordance with local medical insurance policies. They assisted patients from other regions in understanding the regulations for cross-regional medical reimbursement and connected them with online chronic disease management resources to reduce financial strain. Thanks to the above supportive measures, all 25 patients who received staged treatment underwent timely re-intervention, with a 100% follow-up adherence rate. This comprehensive support was associated with improved treatment adherence, better quality of life and smoother rehabilitation.

### 4.4 Limitations and improvement directions of nursing practice

This study is a single-center retrospective analysis with a limited sample size and lack of a control group. In addition, some patients were excluded from the hemodynamic analysis before and after interventional therapy for stenotic vessels due to incomplete paired data. This may reduce the statistical power of this part of the analysis to a certain extent, fail to fully characterize the true hemodynamic changes across all enrolled patients, and introduce potential selection bias. Furthermore, several patients only received follow-up for a few months and thus may not have passed the highest-risk window for restenosis, which means the restenosis rate calculated in this study may underestimate the actual incidence. Therefore, the efficacy of the nursing interventions needs to be further verified through multi-center, prospective studies with prolonged follow-up duration. In addition, PH-FM patients have significant individual differences (such as vascular stenosis classification and comorbidities), and the current personalized adjustment plan for nursing measures needs to be improved. In the future, stratified nursing pathways based on patient classification can be considered, combined with AI technology for complication risk prediction, to further improve nursing accuracy.

## 5 Conclusion

PH-FM is a relatively rare clinical subtype of PH (19), classified as the fifth category (20). Its pathogenesis remains unclear, and there is no radical cure. The preferred treatment is PTPA. Unlike angioplasty in other parts of the body, interventional therapy for PH-FM includes right heart catheterization, transseptal puncture, pulmonary arteriovenous angiography, percutaneous balloon pulmonary arteriovenous angioplasty, and pulmonary arteriovenous stent implantation, which are characterized by technical complexity, high difficulty, and prolonged duration. To ensure the smooth progress of interventional procedure and reduce postoperative complications, perioperative nursing is extremely important. The cases in this study provide practical guidance and reference methods for the nursing of such patients. In the future, it is necessary to continue summarizing relevant experience to form a more standardized nursing plan.

## Data Availability

The original contributions presented in the study are included in the article/Supplementary Material, further inquiries can be directed to the corresponding author/s.
